# Far-Reaching War Activities

**Published:** 1917-09-15

**Authors:** 


### THE WORK OF THE LOCAL GOVERNMENT BOARD.

### Far-reaching War Activities.

The dilettante in official publications might expect the Forty-sixth Annual Report, just issued, to take the form of a massive tome rivalling in bulk and in wealth of statistical detail the volume of returns published annually by the Registrar-General. It is, in fact, a small-paper pamphlet of less than sixty pages, which may be purchased through any bookseller for threepence. The secret of this apparent reticence lies in the facts that, with a view to economy, the Report has been this year further reduced in size by the exclusion of tables, statistics, and other matter, and the great mass of the printed issue of the Board is published in the form of papers presented to Parliament, Stationery Office Publications, Supplements, and Circular Letters. The Report of the Medical Officer of the Board is issued as a supplement, to which again are added in appendices the results of investigations concerning infectious diseases, etc. Johnson said that "knowledge is of two kinds; we know a subject ourselves, or we know where we can find information upon it"; the Local Government Board Report contains in itself little detailed information, but it gives many indications of the existence of such information and as to where it is to be found.

### Provision for Tuberculosis Cases.

With regard to provision for the treatment of tuberculosis, "experience has shown that the accommodation provided in residential institutions is insufficient to meet the demand for beds for advanced cases," and "there is evidence that further provision is needed for the treatment of non-pulmonary tuberculosis both in children and adults." The Board has been able to deal satisfactorily with applications for the treatment of patients not insured under the National Insurance Act who were about to be discharged from the Navy or Army on account of tuberculosis; arrangements have been made, usually by the local authority in whose area the patient resided before enlistment, for institutional treatment to be given. It is well known that the difficulty, above mentioned, of finding accommodation for patients in advanced stages of tuberculosis has arisen in the cases of many discharged soldiers as well as of civilians, and the desirability of ensuring to the former class some resource other than that afforded by Poor-Law institutions has been strongly urged; arrangements are in progress by which it is hoped forthwith to provide the accommodation now required and to increase it in case of need. With regard to the treatment of venereal diseases, in spite of the stress and difficulties of the times, "complete schemes have already been submitted by 115 out of the 145 Councils in England and Wales which are charged with the execution of the Regulations."

The notifications of cases of infectious disease amongst the civilian population are remarkable chiefly in so far as they show, during 1916, a large reduction in the number of cases of fever, especially of scarlet fever; there was also a considerable decrease in the number of cases of diphtheria and erysipelas. Of small-pox 149 cases were notified; a small number, but one in excess of that in any of the preceding three years. In view of the necessity for further facilities for securing the prompt vaccination and re-vaccination of "contacts," medical officers of health have now been empowered to perform vaccination or re-vaccination "on any such persons who are willing. . . ." The bulk of the glycerinated lymph despatched from the Government lymph establishment during the year ended in March 1917 was supplied for the use of the Army, and amounted to a quantity sufficient for 1,565,121 vaccinations or re-vaccinations.

### Work Arising from the War.

Much special work arising out of the war is reported upon. In connection with the administration of the Poor Law it is gratifying to notice the steady continuous decrease in the numbers of casual paupers, a class the elimination of which is possible and desirable. As showing how far-reaching are the activities of the Board it is interesting to note that a regulation has been made by an Order in Council empowering a local authority to grant permission, as a war measure, to keep pigs notwithstanding any by-law to the contrary in force in the district, and that orders have been made in certain towns for the substitution of a calorific standard of gas for an illuminating standard. A standard of calorific power of 500 British thermal units has been prescribed, and an obligation to supply gas at a stated minimum pressure has been imposed.

